# New additions to the cancer precision medicine toolkit

**DOI:** 10.1186/s13073-018-0540-7

**Published:** 2018-04-13

**Authors:** Elaine R. Mardis

**Affiliations:** Institute for Genomic Medicine at Nationwide Children’s Hospital, 575 Children’s Crossroad, Columbus, OH 43205 USA

## Abstract

New computational and database-driven tools are emerging to aid in the interpretation of cancer genomic data as its use becomes more common in clinical evidence-based cancer medicine. Two such open source tools, published recently in *Genome Medicine*, provide important advances to address the clinical cancer genomics data interpretation bottleneck.

## The data paradox in clinical cancer genomics

As the genomic characterization of cancers transitions from a pure research endeavor to a means of providing clinically relevant information for cancer medicine, interpreting the data from next-generation sequencing (NGS)-based clinical assays presents an ongoing challenge [1]. This is a consequence of several realities brought on by more comprehensive testing that is enabled by NGS. At present, most tertiary academic cancer centers in the United States perform an NGS-based test that surveys the known cancer genes, either by sequencing each gene in its entirety or, at a minimum, sequencing the frequently mutated pathogenic sites in these genes. These assays are typically performed for metastatic cancer patients who have failed the standard of care therapy in order to identify one or more alternative therapies that might be available as US Food and Drug Administration (FDA)-approved drugs, or in the context of a clinical trial. Such gene-panel or whole-exome sequencing typically delivers many somatic alterations for each cancer patient tested. Because we do not understand the functional impact of most mutations in cancer genes, assigning causality to specific alterations is quite complex. In addition, the amount of clinical trial- and literature-based information about drug–gene interactions in different cancer types has become overwhelming, so the ability to “keep up” with emergent discoveries is nearly impossible, especially for busy clinicians. In principle, if we expect NGS-based testing to be adopted more broadly in cancer diagnostic medicine, addressing these difficulties in data interpretation is critically important.

Ideally, the output of clinical NGS assays is an annotated diagnostic report that clearly directs the oncologist to the cancer gene-based vulnerabilities of each patient’s tumor, indicated therapies, and clinical trials, and to any other actionable information, such as poor or good prognosis alterations, accompanied by literature-based information to support these assertions. Taken together, intelligently designed computational tools can play an important role in easing the bottleneck at this stage of data interpretation. Such a tool would have two primary functions. First, it would coalesce available data across many patient and tumor samples to build the broadest evidence base for gene variants of unknown functional impact having or lacking causality, as well as identifying known cancer functional alterations in genes. Second, the tool would organize and present this gene-specific analysis of known and predicted functional impact with accompanying current information on therapeutic-, literature-, and clinical trial-based annotations—in essence, the diagnostic report.

## An expanded toolkit for research and the clinic

Recently, *Genome Medicine* published two studies describing such tools, each developed independently by two different groups but aimed at the same goals. Each tool has unique aspects that may indeed facilitate the interpretation bottleneck of NGS-based cancer diagnostics. One article describes the Cancer Genome Interpreter (CGI) tool [2] developed under the leadership of Nuria Lopez-Bigas, whose group has previously contributed important tools that predict cancer variant functionality [3] and identify cancer driver genes [4, 5]. The CGI tool is based on a set of “catalogs” that contain (1) known cancer driver genes, (2) validated alterations of these genes that are known to contribute to cancer onset or progression, (3) a curated database of biomarkers of cancer drug response, and (4) a compendium of small-molecule drug–gene interactions. In addition, CGI has a bioinformatics-based predictor of functionality for unknown variants in genes that identifies those alterations that are most likely to contribute to cancer, so they can be interpreted alongside known cancer driver alterations. CGI uses these catalogs and its functional predictor to systematize the interpretation of cancer genomes by identifying all known and likely tumorigenic alterations, including variants with unknown functional impact, and then annotates those variants that constitute biomarkers of drug response and organizes them according to distinct levels of clinical evidence. At its essence, the main utility of CGI is to direct attention to known and predicted cancer drivers for the consideration of therapeutic indications or prognosis, as appropriate, based on the data available in its catalogs.

The second article describes a tool, MTB report, from Tim Beißbarth and colleagues [6], that automatically matches cancer patient-specific genomic alterations to treatment options based on support from the literature, clinical trials, and publicly available databases as a means of facilitating the use of NGS in clinical practice. This tool includes information about off-label therapy use (i.e., approved therapies for a specific target that have not yet been approved for the tissue site in the patient being assayed) that may be applicable in advanced-stage patients, thereby expanding the treatment options delivered in the report it produces. This information is delivered as a six-level system that ranks variant–drug associations according to the strength of evidence for each drug as determined by (1) evidence of activity in cancer type, and (2) evidence of drug approval or clinical trial status (including preclinical studies). The resulting tool was evaluated using publically available patient data obtained from The Cancer Genome Atlas (TCGA) [7] and the American Association for Cancer Research (AACR)‘s Project Genomics Evidence Neoplasia Information Exchange (GENIE) [8], then applied as a proof of concept to the analysis of 11 cancer cases from the Nationales Centrum Für Tumorerkrankungen (NCT) Molecularly Aided Stratification for Tumor Eradication (MASTER) trial. In the latter evaluation, concordance was high but not perfect, mainly due to some information lacking in the MTB report tool-associated databases, which will no doubt improve over time and with end-user feedback.

## Future prospects: progress through data sharing

Importantly, both groups have made their source code available publicly, enabling uptake and customization of the tools by many institutions and researchers. This availability is to be championed, as is open sharing of data, software source code, and databases that enhance the performance, breadth, and accuracy of these tools in this rapidly advancing field. Another strength of both tools is the consideration of multiple types of DNA alterations (beyond point mutations), since large-scale genomic characterization studies have fully demonstrated that all types of alterations can contribute to cancer development [9]. Additional sophistication in these tools will result from the inclusion of RNA-based expression-level data from unbiased RNA-sequencing or targeted RNA-sequencing approaches in the interpretation of genes and therapeutics, especially in light of the support it may provide for amplified genes as cancer drivers.

These two reports are also illustrative of the fact that the terminology characterizing cancer gene alterations and their interpretation are in need of clearer, standardized definitions for publication. Adopting a defined terminology around descriptors such as “actionable” and “targetable”, among others, will make reports of diagnostic yield from NGS-based cancer diagnostic assays more comparable when evaluating both assays and interpretive tools. So far, we have guidelines regarding evidence tiers by which links between genomic data and their role in cancer can be reported, as provided by regulatory bodies that oversee the offerors of these tests [10]. While these are an important first step, there is no common set of descriptors to characterize the results of cancer NGS test findings relative to therapeutic indications. As such, establishing the clinical utility or benefit from such testing becomes subjective.

In summary, computational interpretation tools such as those described here mark important steps forward in expanding the use of NGS-based assays for cancer medicine, because they address the challenges of data interpretation. Ideally, these tools will be tested and applied by cancer care providers (oncologists in particular) across cancer care organizations, regardless of whether the corresponding NGS assay is performed on site, or as a send-out test. This implementation will help to evaluate the extent to which these tools facilitate NGS-based assay interpretation and, importantly, could enable more wide-scale access to such testing. Ultimately, these and related efforts will not only dissolve barriers to access but also will reinforce the clinical benefit that patients receive from precision cancer medicine, so that reimbursement for these tests from insurance providers becomes routine. As a consequence, NGS testing will become the standard of care, adding precision to each patient’s diagnosis and treatment.

## Abbreviations

AACR: American Association for Cancer Research
CGI: Cancer Genome Interpreter
GENIE: Genomics Evidence Neoplasia Information Exchange
MASTER: Molecularly Aided Stratification for Tumor Eradication
NCT: Nationales Centrum Für Tumorerkrankungen
NGS: Next-generation sequencing
TCGA: The Cancer Genome Atlas
